# Pomegranate Seed Oil and Bitter Melon Extract Affect Fatty Acids Composition and Metabolism in Hepatic Tissue in Rats

**DOI:** 10.3390/molecules25225232

**Published:** 2020-11-10

**Authors:** Agnieszka Stawarska, Tomasz Lepionka, Agnieszka Białek, Martyna Gawryjołek, Barbara Bobrowska-Korczak

**Affiliations:** Department of Bromatology, Faculty of Pharmacy, Medical University of Warsaw, Banacha 1, 02-097 Warsaw, Poland; tomasz.lepionka@wihe.pl (T.L.); a.bialek@ighz.pl (A.B.); martyna_gawryjolek@o2.pl (M.G.); barbara.bobrowska@wum.edu.pl (B.B.-K.)

**Keywords:** pomegranate seed oil, bitter melon, fatty acids, prostaglandin E_2_, Δ^6^ and Δ^5^-desaturase

## Abstract

Pomegranate seed oil (PSO) and bitter melon dried fruits (BME) are used as natural remedies in folk medicine and as dietary supplements. However, the exact mechanism of their beneficial action is not known. The aim of study was to assess how the diet supplementation with PSO and/or with an aqueous solution of *Momordica charantia* affects the metabolism of fatty acids, fatty acids composition and the level of prostaglandin E_2_ (PGE_2_) in rat liver. Animals (Sprague-Dawley female rats, *n* = 48) were divide into four equinumerous groups and fed as a control diet or experimental diets supplemented with PSO, BME or both PSO and BME for 21 weeks. Fatty acids were determined using gas chromatography with flame ionization detection. PSO added to the diet increased the rumenic acid content (*p* < 0.0001) and increased accumulation of *n*-6 fatty acids (*p* = 0.0001) in hepatic tissue. Enrichment of the diet either with PSO or with BME reduced the activity of Δ^6^-desaturase (D6D) (*p* = 0.0019), whereas the combination of those dietary factors only slightly increased the effect. Applied dietary supplements significantly reduced the PGE_2_ level (*p* = 0.0021). No significant intensification of the influence on the investigated parameters resulted from combined application of PSO and BME. PSO and BME have potential health-promoting properties because they influence fatty acids composition and exhibit an inhibiting effect on the activity of desaturases and thus they contribute to the reduction in the metabolites of arachidonic acid (especially PGE_2_).

## 1. Introduction

It is widely known that plant products being the components of varied diet can play a role in the prevention and treatment of many diseases. They contain various bioactive compounds which possess enormous medicinal value. However, the exact mechanisms of their action are often unknown.

*Punica granatum* L. (Lythraceae) is one of the oldest edible fruits which has a long history as a medicinal fruit. Since antiquity, pomegranate has been known for its numerous therapeutic properties that influence health. It has been used in traditional folk medicine in Asia, South America and East Africa [1]. Recently, results of studies indicate the beneficial properties of pomegranate seed oil (PSO), which has been linked with treatment and prevention of cancer, cardiovascular disease, Alzheimer’s disease and diabetes [2,3,4,5,6,7]. Current research indicates that the most therapeutical PSO constituent is the beneficial fatty acids (FA) composition. Low concentrations of saturated and monounsaturated FA (SFA and MUFA, respectively) and high content of polyunsaturated FA (PUFA) make it desirable in the human diet [8,9]. PSO exerts a hypoglycemic effect, which may result from the presence of punicic acid (PA)—one of the conjugated linolenic acids (CLnA) [3,4]. PSO as a rich source of PUFA is especially susceptible for lipid peroxidation, as previously indicated [9], which justifies the need of its coupling with antioxidant agents in diet.

*Momordica charantia* L. (Cucurbitaceae) is commonly known as bitter melon, bitter apple, bitter cucumber, bitter gourd, balsam pear or karela and is widely cultivated in tropical areas including parts of Asia, Africa, South America and Caribbean [10,11]. Compounds isolated from the fruit and seeds of bitter melon plant that are believed to contribute to its hypoglycemic activity include charantin (a steroid glycoside) and polypeptide “p” or plant insulin (a 166-residue insulin mimetic peptide). Bitter melon is also known to contain additional glycosides such as mormordin, vitamin C, carotenoids, flavanoids, polyphenols and α-eleostearic acid [11,12,13]. Bitter melon is extensively used in Chinese and Indian medicine as anti-diabetic, anti-inflammatory, anti-microbial, anti-tumor, anti-leukemic [10] but fruits are also consumed as a foodstuff. Dried fruits of bitter melon are available as bitter melon ‘tea’ ready to prepare the bitter melon fruits aqueous extract. This is the most common manner of bitter melon fruits consumption.

PSO and bitter melon fruits are used not only in traditional folk medicine but also as popular dietary supplements, which are recommended as anti-atherogenic and anti-carcinogenic agents. In present experiment combined use of PSO and bitter melon extract (BME) is be justified by the antioxidant properties of BME which has been shown previously [14] and also is to reflect the multi-supplementation of diet with PSO and bitter melon. The exact mechanism of their action is under our investigation but one of the proposed pathways is their modulation of fatty acids hepatic metabolism.

Liver plays an essential role in lipid metabolism. Bile, which facilitates the digestion and intestinal absorption of lipids, is produced by the liver. Moreover, this organ integrates the synthesis and metabolism of plasma lipoproteins. Moreover, the fatty acid synthesis and oxidation take place in liver, as well as the formation of ketone bodies from fatty acids. Transformation of fatty acids in hepatic tissue depends, among others, from the activity of desaturases, which are pivotal enzymes involved in fatty acids hepatic metabolism. Desaturases can insert double bonds into different positions of carbon chain, but not higher than Δ^9^. Their action is fundamental for metabolic routes of *n*-3 and *n*-6 fatty acids, but also for metabolic transformations of conjugated linoleic acids (CLA). Our previous results revealed that the neoplastic process has a stimulating effect on the activity of Δ^6^-desaturase, whereas presence of CLA in the animals’ diet decreased Δ^6^-desaturase activity, which is one of mechanisms of anticarcinogenic activity of CLA [15]. Many studies demonstrated relationship between desaturase activity and certain diseases such as insulin resistance, coronary artery disease and obesity. The results are often inconclusive and require further research. However, new components of the diet that will positively affect the metabolism of fatty acids are still being sought. We previously indicated, that dietary supplementation with PSO and/or BME increased the CLA concentration in serum of rats in physiological conditions [14]. The obtained results lead us to in-depth research on the influence of PSO and BME on fatty acid liver metabolism.

The exact aim of this study was to assess how the dietary supplementation with PSO and/or BME affects the fatty acids composition in the liver and its microsomal fraction (fraction responsible for fatty acids metabolism). The levels of PGE_2_ and the activity of key enzymes catalyzing lipids metabolism (desaturases) were measured. It was also examined whether or not a joint supplementation intensified the effect.

## 2. Results

### 2.1. Fatty Acids Profile and Characteristics of Applied Dietary Supplements

**Figure 1:**
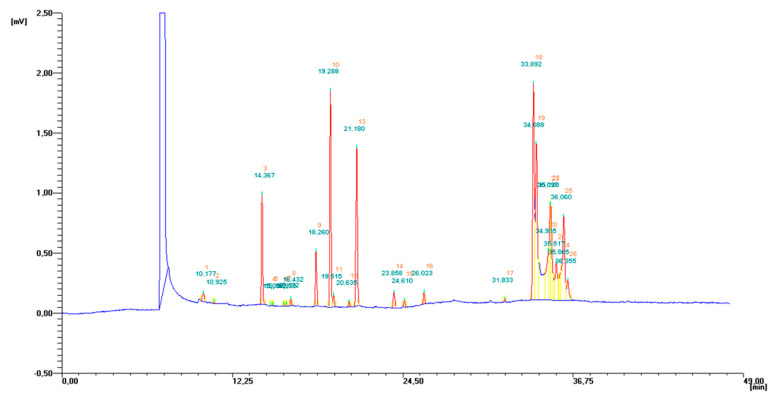
GC-FID chromatogram of PSO.

Fatty acids profiles [%] of applied dietary supplements administrated to rats are given in Table 1. Fatty acids profile were determined using gas chromatography with flame ionization detection. Exemplary GC-FID chromatogram of PSO is given in Figure 1.

In applied PSO palmitic, linoleic, stearic and α-linolenic acid acids predominated whereas punicic acid constituted only 6.6 ± 0.2% of all FA. The examination of the FA profile in BME confirmed small amounts of FA, of which linoleic, palmitic and oleic acids acid were determined as predominating. Moreover, some other FA acids were present in examined supplements, but their identification was impossible because of the lack of proper standards. As previously explained [14], in BME, chlorogenic acid was also determined in relatively high amounts whereas other phenolic acid derivatives and flavonoids were detected in trace amounts. The antioxidant and antiradical activity of BME measured in DPPH and FRAP assays revealed its rather small potency [14].

### 2.2. Liver Weight and Fat Content in Experimental Groups

**Figure 2:**
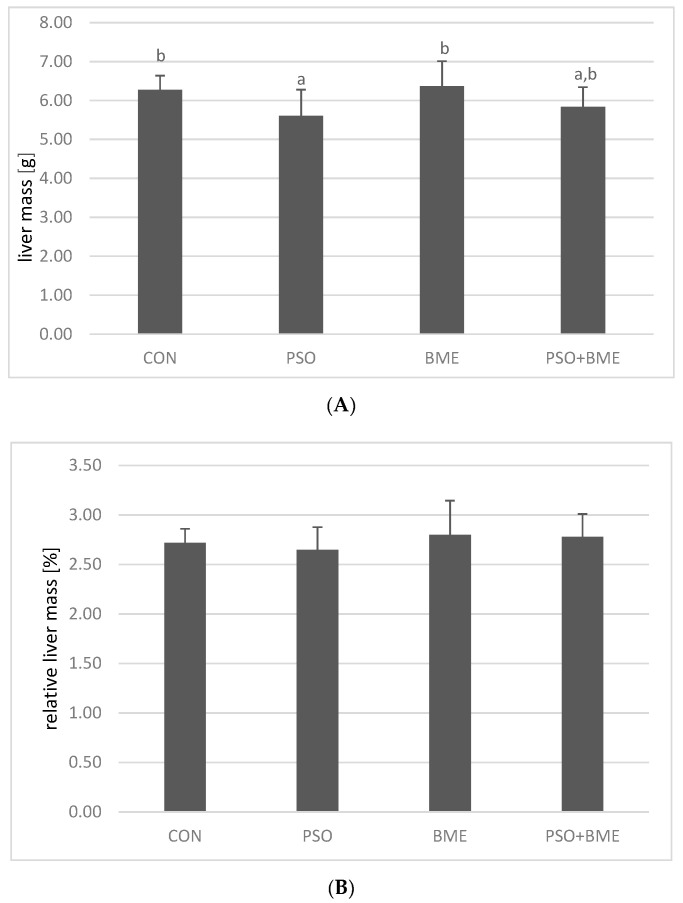

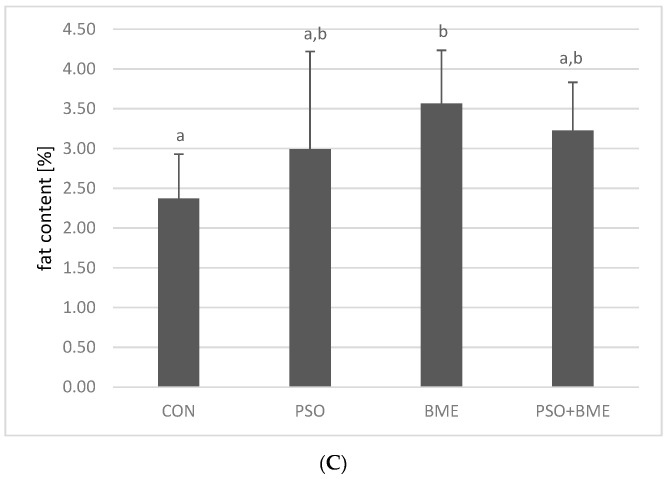
Liver mass (**A**), relative liver mass (**B**) and fat content [%] (**C**) in rats of experimental groups. CON—control group; PSO—group receiving PSO in the amount of 0.15 mL/day; BME—group receiving BME ad libitum; PSO + BME—group receiving PSO in the amount of 0.15 mL/day and BME ad libitum. All data are shown as mean values ± standard deviation of data obtained from all animals (*n* = 12) in each group. Values with a different letter index are significantly different from each other (*p* ≤ 0.05) in post-hoc RIR Tukey test.

No differences in food and fluid intake were observed among the investigated groups (data not shown) but applied dietary modification influenced liver mass and fat content, without any impact on relative liver mass (Figure 2). PSO supplementation decreased liver mass, as mean liver mass in the PSO group was significantly (*p* = 0.0042) lower than in CON and BME groups (Figure 2A). However, this dependence was not reflected in relative liver mass (*p* > 0.05) (Figure 2B). Results concerning body weight have been given previously [14]. The introduction of BME into the diet of rats significantly increased fat content in the livers of the BME group (*p* = 0.0069), and in case of PSO, which was an additional dietary fat source incorporated into the diet of PSO and PSO + BME groups, similar tendency was observed (Figure 2C).

### 2.3. Fatty Acids Profile in Liver

**Figure 3:**
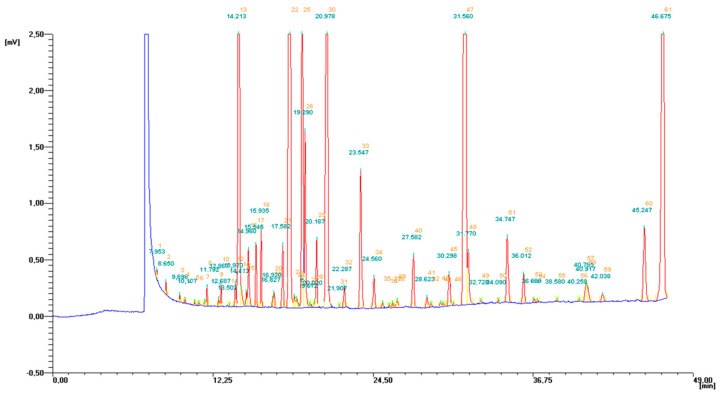
Exemplary GC-FID chromatogram of fatty acid profile in liver.

In the lipid fraction extracted from the liver samples, we have detected and identified 30 FA (Figure 3), and we estimated the percentage share of each fatty acid in the total fatty acids’ pool (Table 2). The fatty acid profile in liver of experimental animals, diversified according to applied dietary supplementation, is shown in Table 2. The saturated fatty acids (SFA) share in the total fatty acids pool was significant, as it constituted more than 40% of all fatty acids in each dietary group. Among all SFA, palmitic and stearic acids were present in the highest amount, and we did not observe any difference in their share between groups (Table 2). Dietary supplementation with PSO resulted in a decrease in the content of pentadecanoic (C15:0), heptadecanoic (C17:0) and heneicosanoic (C21:0) fatty acids in the PSO group in comparison to the CON group. We observed similar effects in the PSO + BME group. In the BME group effects were predominantly opposed, as the contents of lauric (C12:0) and myristic (C14:0) acids were lower, and heptadecanoic (C17:0) share was higher in comparison to the PSO group. Despite some differences in the SFA profile, there was no difference in the total SFA content among the groups. As far as monounsaturated fatty acids (MUFA) are concerned, except oleic acid (C18:1 *n*-9 *cis*), they were present in liver of the experimental animals in rather small amounts (Table 2). MUFA constituted about 8% of the total fatty acid pool in the all groups, and we did not observe any difference in their share among groups. Dietary supplementation with PSO resulted in the lowest level of elaidic acid (C18:1 *n*-9 *trans*) in the PSO group. PSO administration resulted in the lowering share of heptadecenoic acid (C17:1), but significant difference was observed only in the PSO + BME group. The polyunsaturated fatty acid (PUFA) share in the total fatty acids profile was significant, as they constituted 43.5–45.5% of all fatty acids, depending on the experimental group. We observed significantly higher levels of the total PUFA content in the groups PSO and PSO + BME in comparison with the CON group (Table 2). As far as *n*-3 PUFA are concerned, simultaneous administration of PSO and BME resulted in the elevation of eicosatrienoic acid (C20:3 *n*-3) in relation to the PSO and CON group. On the contrary, applied dietary supplementation resulted in lower docosahexaenoic (C22:6 *n*-3) levels in all experimental groups compared with the CON group, but only PSO effect was statistically significant. As far as *n*-6 PUFA are concerned, we did not observe one tendency for all examined fatty acids. PSO significantly increased GLA (C18:3 *n*-6) in the total fatty acids pool. On the contrary, dietary supplementation with BME resulted in considerably higher levels of C20:3 *n*-6 fatty acid, but it decreased levels of C18:2 *n*-6 *trans* in the PSO + BME group (statistically significant). Regarding AA (C20:4 *n*-6), we observed a statistically significant elevated share in the livers of all experimental groups in relation to the CON group (Table 1). We did not observe any differences in the levels of linolenic acid among the experimental groups. Regarding conjugated fatty acids, no CLnA isomers were detected in any of the experimental groups, while one of the CLA isomers—rumenic acid (RA), was detected in liver of all groups. RA content in the groups supplemented with PSO, which did not contain any CLA isomers, significantly exceeded its content in both CON and BME group, where its share was vestigial. What is more, the RA content tends to increase in the group PSO + BME compared with the PSO group. As far as a total percentage share of *n*-3 PUFA, we observed that applied supplementation tends to reduce the *n*-3 content in the all experimental groups in relation to CON, but only in the PSO group was this difference statistically significant. Conversely, both PSO and BME seem to significantly increase the total share of *n*-6 PUFA in comparison with the CON group. The *n*-6/*n*-3 ratio was also determined; we observed, that applied supplementation resulted in a statistically significant increase in the *n*-6/*n*-3 ratio in all experimental groups.

### 2.4. Fatty Acids Profile in Hepatic Microsomes

**Figure 4:**
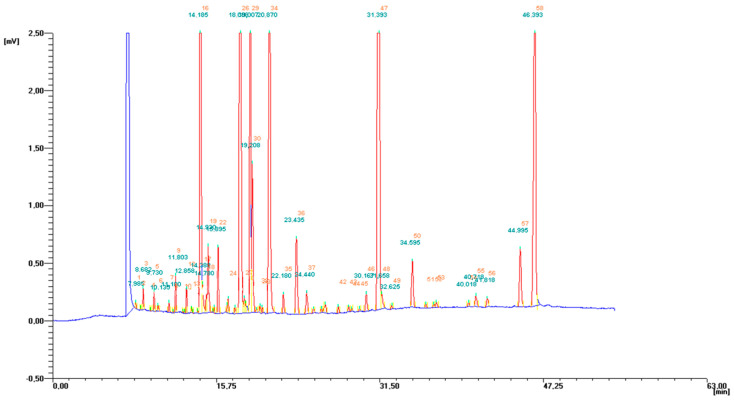
Exemplary GC-FID chromatogram of fatty acid profile in microsomal fraction.

In the examined samples of hepatic microsomes, 30 fatty acids, including 11 SFA, 7 MUFA, and 12 PUFA (Figure 4), were detected and determined (Table 3). SFA constituted about 44% all fatty acids in the fatty acids’ pool, and we did not observe any differences between their total content between groups. Among SFA only two: palmitic (C16:0) and stearic (C18:0) were characterized by higher contents, but none of them showed any significant difference in concentration between experimental groups (Table 3). Diet supplementation with PSO significantly reduced the percentage share of pentadecaenoic acid (C15:0) in group PSO in relation to CON group (Table 3). Simultaneous administration of PSO and BME resulted in lowering the share of heptadecaenoic acid (C17:0) in all experimental groups except CON group. Moreover, that diet modification, significantly lowered content of lauric acid (C12:0) in group PSO + BME compared with groups PSO and BME. On the contrary, the applied diet modification seems to increase the percentage share of tricosanoic acid (C23:0) in all experimental groups except CON group, but statistically significant differences were detected only in group PSO + BME.

As MUFA are concerned, their content ranged from 6 to 8% of the total fatty acids’ pool. We observed that introduction of the PSO in diet resulted in a statistically significant decrease in the overall content of MUFA in PSO and PSO + BME groups. Moreover, the diet modification resulted in lowering the share heptadecenoic acid (C17:1), elaidic acid (C18:1 *n*-9 *trans*) and oleic acid (C18:1 *n*-9 *cis*) in comparison to CON group (Table 2). Simultaneous administration of both diet supplements seems to reduce the content of C20:1 fatty acid in group PSO + BME compared to CON and BME group. Moreover, diet supplementation with PSO and BME seems to result in lowering share of C14:1 in all experimental groups in comparison with CON group.

PUFA constituted from 41 to 44% of the total fatty acids’ pool. The lowest total amount of PUFA was observed in CON group, which was significantly lower than PUFA content in the PSO and BME groups. We observed differences in concentrations for four fatty acids. Diet supplementation with PSO and BME significantly increased the share of *n*-6 PUFA, as the concentrations of GLA (C18:3 *n*-6) and AA (C20:4 *n*-6) in groups PSO, BME and PSO + BME were higher in comparison to CON group (Table 3). On the contrary, diet modification resulted in lowering of the share of C20:2 in all supplemented groups in relation to CON. As far as conjugated fatty acids are concerned, we did not detect any isomer of CLnA in any experimental group. Furthermore, one of the CLA isomers—RA—was detected in all experimental groups. Addition of PSO to the animals’ diet resulted in a significantly higher concentration of RA in groups PSO and PSO + BME referring to CON and BME group.

Concerning the total percentage share of *n*-3 PUFA, we did not observe any statistically significant difference between groups. Conversely, both PSO and BME seem to significantly increase total share of *n*-6 PUFA in relation to the CON group. As far as the *n*-6/*n*-3 ratio is concerned, we observed, that diet supplementation in groups PSO and BME resulted in a statistically significant increase in the ratio in comparison to CON group. Moreover, the PUFA/SFA ratio and peroxidability index were determined; diet supplementation with PSO and bitter melon aqueous extract significantly increased the PUFA/SFA ratio in all supplemented groups, as well as it increased the PI in groups PSO and BME in relation to CON group.

### 2.5. Analysis of Δ^6^- (D6D) and Δ^5^-Desaturase (D5D) Activities

**Figure 5:**
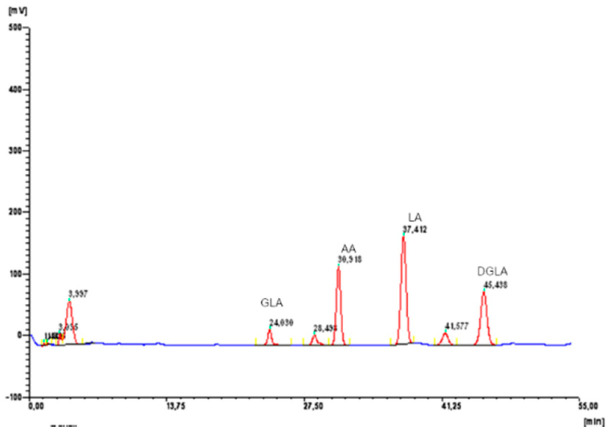
Exemplary HPLC chromatogram of unsaturated fatty acids: GLA, LA, AA, DGLA in microsomal fraction.

Desaturase activities were determined indirectly and expressed as an increase in AA concentration after incubation in conditions similar to those observed in vivo. D6D and D5D indices were also calculated: D6D index as GLA/LA concentration ratio and D5D index as AA/DGLA concentration ratio (Figure 5).

**Figure 6:**
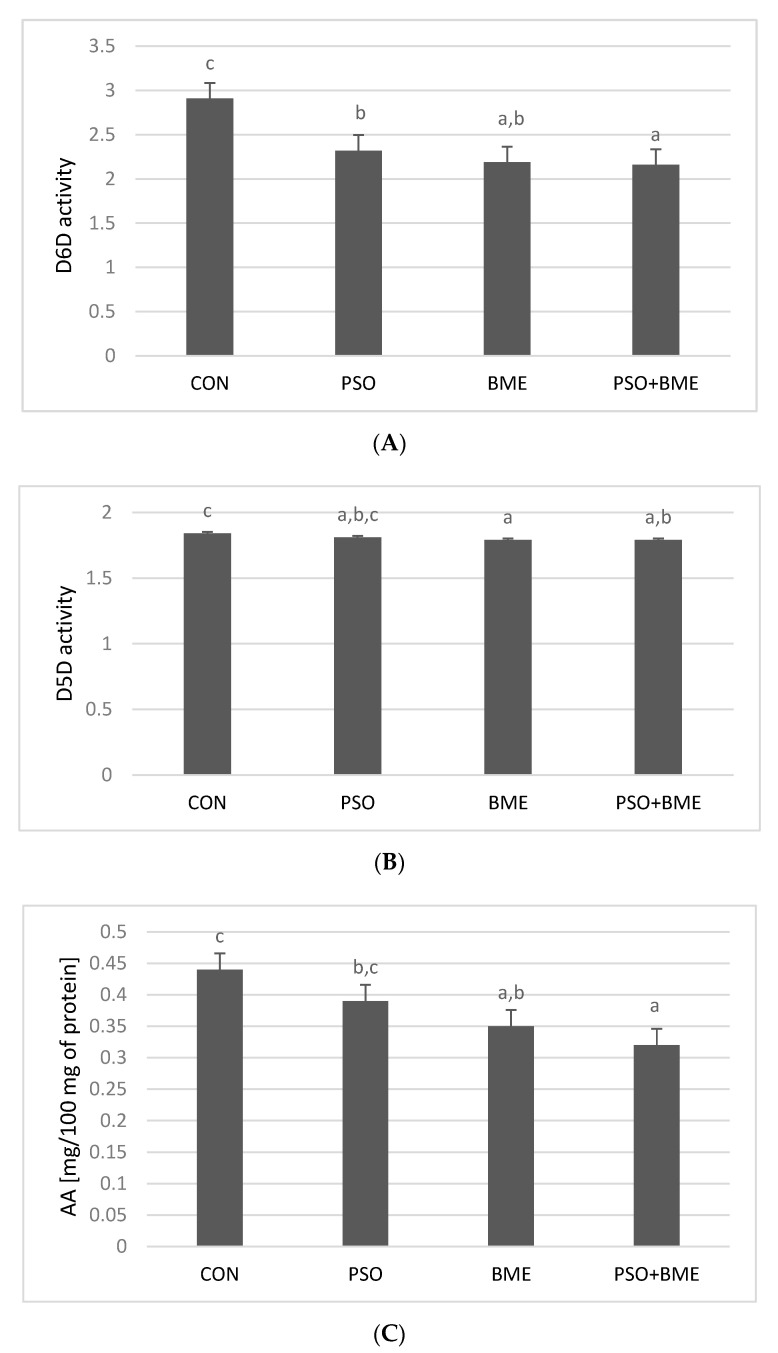
Activities of D6D (**A**) and D5D (**B**) in hepatic microsomes of experimental groups. AA concentration increase (mg/100 mg protein) in rat hepatic microsomes (**C**). CON—control group; PSO—group receiving PSO in the amount of 0.15 mL/day; BME—group receiving BME ad libitum; PSO + BME—group receiving PSO in the amount of 0.15 mL/day and BME ad libitum. All data are shown as mean values ± standard deviation of data obtained from all animals (*n* = 12) in each group. Values with a different letter index in one row are significantly different from each other (*p* ≤ 0.05) in post-hoc RIR Tukey test or multiple comparison test. D6D—Δ^6^-desaturase index (×10^−3^), D5D—Δ^5^-desaturase index.

The highest AA increase (~0.44 mg/100 mg protein) was observed in CON group, whereas the lowest in the PSO + BME group (~0.32 mg/100 mg protein) (Figure 6C). The highest D6D index, expressing the enzyme activity, was observed in microsomes obtained from CON group (2.91 × 10^−3^) (Figure 6A). Conversely, both PSO and BME significantly decreased D6D index in comparison with the CON group. In the case of D5D activity, its value was significantly lower in comparison with the CON group only in the BME and PSO + BME groups (Figure 6B). The supplementation with PSO did not significantly affect the activity of the examined enzyme.

### 2.6. Liver PGE_2_ Levels

**Figure 7:**
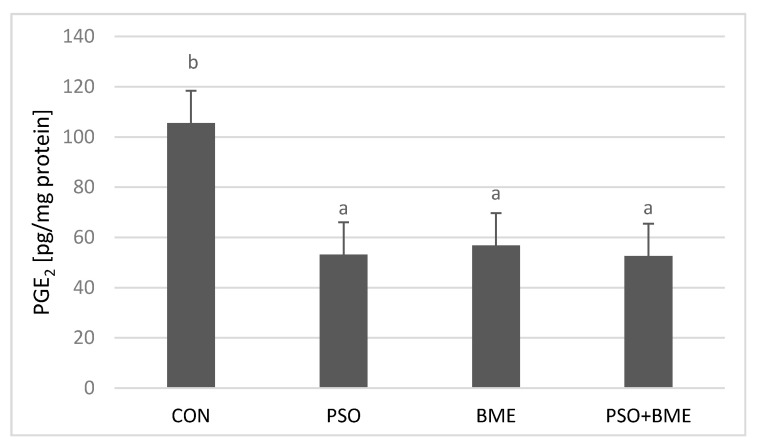
PGE_2_ (pg/mg protein) content in liver of experimental groups. CON—control group; PSO—group receiving PSO in the amount of 0.15 mL/day; BME—group receiving BME ad libitum; PSO + BME—group receiving PSO in the amount of 0.15 mL/day and BME ad libitum. All data are shown as mean values ± standard deviation of data obtained from all animals (*n* = 12) in each group. Values with a different letter index in one row are significantly different from each other (*p* ≤ 0.05) in post-hoc RIR Tukey test.

The level of PGE_2_ as expressed in pg/mg protein is presented in Figure 7. On the basis of the presented results it was found that there is a statistically significant difference in the concentration of PGE_2_ between particular groups (*p* = 0.0021). The highest level of PGE_2_ was obtained in the CON group (105.55 ± 27.42 pg/mg protein) whereas the lowest concentration was obtained in the PSO + BME group (52.62 ± 13.86 pg/mg protein). The results obtained from a single supplementation, either with PSO or with BME, did not differ significantly from those obtained in the PSO + BME group; but, each time, they were much lower than the concentrations obtained in the CON group.

## 3. Discussion

Results obtained revealed that PSO decreased liver mass (Figure 2A) and tend to increase the fat content in liver (Figure 2C), whereas BME appeared to increase the fat content in liver in the most potent way (Figure 2C).

Total SFA and MUFA content did not differ among dietary groups whereas significant differences concerning individual SFA or MUFA levels were observed. PSO increase PUFA and decreased *n*-3 PUFA in liver whereas both dietary supplements increased *n*-6 PUFA share. However, changes observed in individual fatty acids share did not influence PI (Table 2).

In the case of the microsomal fraction of hepatic tissue, PSO decreased MUFA share, whereas both applied supplements increased *n*-6 PUFA content without any impact on *n*-3 PUFA, which resulted in increase in PI in all supplemented groups (Table 3).

The nature of hepatocyte cell membranes and their microsomal fraction is slightly different. Microsomes are concerned as vesicles of the hepatocyte endoplasmic reticulum and they are heterogenous vesicle-like artifacts (~20–200 nm diameter) re-formed from pieces of the endoplasmic reticulum when eukaryotic cells are broken-up in the laboratory. They contain several microsomal enzymes, including flavin monooxygenases, cytochrome P450, NADPH cytochrome c reductase, UDP glucoronosyl transferases, glutathione-S-transferases, epoxide hydrolases and desaturases. The fatty acids composition of endoplasmic reticulum is different than the fatty acids composition of hepatocytes cell membranes. It can also be modified by the diet, which, in turn, can influence the activity of microsomal enzymes, which was evaluated in present paper.

The applied supplementation causes decrease in the activity of D6D (Figure 6A), which corresponded with the decrease in PGE_2_ content in all experimental groups receiving diet supplementation (Figure 7).

Investigated botanicals gain increasing interest worldwide, not only as folk medicines but also as dietary supplements of no harmful effects. Different in vitro and in vivo toxicity test revealed PSO as rather non-toxic and safe [16]. As far as bitter melon is concerned, the potential use of these fruits was summarized and evidenced that in most of the experimental models their consumption is safe with no adverse effects [16,17,18].

PSO gained special attention as being health-promoting, due to the beneficial fatty acids composition. The low contents of SFA and MUFA and the high content of PUFA, among which the presence of PA—one of conjugated linolenic acids (CLnA) isomers made it interesting as potential dietary supplement [19]. However, as revealed before, the quality of PSO offered as dietary supplements differ [9]. In PSO applied to animals in the present experiment, SFA constituted ~40% of FA whereas MUFA hare was 11% and identified PUFA constituted only, e.g., 36%, with some of the FA being unidentified. Thorough analysis of chromatograms may us to assume that they may be other CLnA isomers, which identification was impossible because of the lack of proper analytical standards. Results of numerous studies reveal beneficial preventive properties of PSO in different pathological states, e.g., diabetes, cancer, hyperlipidemia [20]. Conjugated fatty acids are widely investigated as main bioactive components of PSO and many possible mechanisms of their action are proposed, among which competition with PUFA in metabolic pathways is of great interest. We previously revealed that CLA can compete with PUFA and influence their concentration and the concentration of their metabolites in serum and in mammary tumors [21,22,23] and in liver as well as the activity of enzymes participating in PUFA metabolism [24,25]. As a natural continuation of these findings, we investigated whether PSO, as a rich source of CLnA, can also act in similar way.

Results obtained in the present study did not confirm competition of CLnA from PSO with PUFA. Applied supplementation can influence oxidation processes as we observed elevated values of PI in liver microsomes of all examined groups. We did not detect PA, neither in the microsomal fraction nor in the whole hepatic tissues of any examined group of rats, although in the serum of the PSO group, PA was previously detected [26]. RA was present in all examined samples and its share was elevated in two groups supplemented with PSO. Our observations are in accordance with those of Yamasaki et al. [27] as they detected dose-dependent accumulation of RA in serum, liver and adipose tissue of mice fed diet supplemented with increasing levels of PSO. As previously suggested by Tsuzuki et al. [28] most of the PA given to rats is absorbed in the intestine within 24 h and part of this fatty acid is converted to RA. This Δ-13 saturation reaction depends on NADPH and occurs not only in rat intestine but also in liver and kidneys [29]. Obtained results seem to confirm this conversion of PA into RA and preferential accumulation of RA in hepatic tissue of rats. Moreover, our observation made for hepatic tissue and its microsomal fraction differ from our previous observation made for serum as slightly elevated levels of RA were observed in serum of rats supplemented solely with PSO (PSO group) in relation to CON group whereas the highest share of RA in total fatty acids pool was detected in group receiving solely BME (BME group) [21]. One of the mechanisms of CLnA action seems to be the possibility of its transformation into CLA. Tsuzuki et al. [29] proved that after a 4-week supplementation of rats with 1% α-eleostearic acid, the *cis*-9, *trans*-11 CLA acid appeared in the rats’ liver and plasma. Similar results were obtained in the case of PA which is metabolized to *cis*-9, *trans*-11 CLA [30]. In the liver, plasma, kidneys, heart, brain and adipose tissue of the examined rats, a CLA isomer was identified. Hence, it was hypothesized that the health-promoting properties of CLnA can be associated with the activity of CLA. Our earlier investigations showed that the supplementation with CLA had an inhibiting effect on the activity of desaturases [25]. The supplementation with PSO also affected the activity of the examined enzymes. Enrichment of the diet either with PSO or BME reduced the activity of D6D, whereas the combination of those dietary factors only slightly increased the effect. Moreover, in the case of D5D, enzyme inhibition was found, although it was statistically significant only for the BME and PSO + BME groups. A similar tendency was observed as concerns the increase in AA. In animal studies, the activity of D6D positively correlates with insulin resistance and obesity. In contrast, enrichment of the diet with PUFAs reduces the expression of D6D [31]. There is an evidence that the conjugated linolenic acids exhibit potent anti-inflammatory activity, which results both from a decrease in AA transformation and the inhibition of pro-inflammatory cytokines such as TNF-α, or IL-6 [32]. Mashhadi et al. [33] showed the reduction in the PGE_2_ level as a result of CLnA treatment. Those authors proved that the above-mentioned dependence is related to the inhibition of both COX-1 and COX-2. On the other hand, Wang et al. [34] revealed that CLA not only inhibits the formation of COX-2 but also contributes to a decrease in the number of EP_2_ receptors for PGE_2_, which leads to the reduction in PGE_2_ concentration and thus increases the chances of recovering from cancer. On the basis of the obtained results the hypothesis about the effect of PSO-enriched diet on the formation of pro-inflammatory PGE_2_ was confirmed. In comparison with the control group, in the liver of rats that were supplemented with BME, the PSO, and the combination of both, a significantly lower level of the examined prostaglandin was observed. No statistically significant difference in mean concentrations of PGE_2_ was observed between the group that was supplemented with BME and the group that received PSO. The aim of a combined supplementation was to determine whether or not the potential effects of both products sum up. The obtained results showed that there is no such dependence because the mean level of PGE_2_ in the animals from the PSO + BME group did not differ from the mean values obtained for the remaining groups supplemented with dietary supplements. The influence of applied botanicals was observed in case of lipid profile of hepatic tissue, especially of its microsomal fraction, and also in case of enzymes involved in fatty acids metabolism, as well as in case of AA metabolite—PGE_2_. Both botanicals appeared to influence body lipid metabolism, and in some cases (e.g., desaturases activity and PGE_2_ content) their action was similar, whereas in case of fatty acids, their impact was diversified, both similar and opposite, and not as much pronounced as in case of PGE_2_. It may result from the fact, that applied supplementation slightly changed the over fatty acids intake. In case of PSO this impact was more evident than in case of BME. In BME, which was an aqueous extract, the content of fatty acids was very small and changes in fatty acids profile in BME group may result from the influence of other bioactive compounds on body lipid metabolism. Moreover, the influence of PSO was not limited to increase fatty acid intake, but also resulted from the presence of other bioactive compounds. Lack of synergistic effect of combined PSO and BME supplementation in case some examined variables may rather indicate their antagonistic influence. To summarize, the obtained results clearly show that proper dietary supplementation is a rather complex issue as simultaneous intake of even two dietary supplements, especially those of botanical origin, which are complex mixtures of many bioactive compounds, may result in their interactions and distinctly modify their influence on the organism.

## 4. Materials and Methods

### 4.1. Pomegranate Seed Oil

Commercially available cold pressed, unrefined oil from seeds of pomegranate fruits (INCI: Punica Granatum (Pomegranate) Seed Oil, Zielony Klub) was purchased from the local market (Kielce, Poland) with expiration date ≥6 months. It was stored at 8 °C before administration to animals and it was given to animals within its expiration date.

### 4.2. Bitter Melon Aqueous Extract

Commercially available dried fruit of bitter melon (*Momordica charantia*) (Tra Kho Qua, Gohyah Tea, CTE JSCO) was purchased from a local grocery store in Warsaw, Poland. Fresh aqueous extracts (tea—BME) were prepared daily according to the manufacturer’s description. Briefly, water (80 °C) was added to a weighed amount of dried fruits to obtain 2% (*w*/*v*) extract. After 10 min, the extract was filtered and freshly cooled tea was given to the animals daily. The concentration and the method of tea preparation were in accordance with the manufacturer’s recommendation for this dietary supplement. The detailed characteristic of BME was given previously [14].

### 4.3. Animals

Performed animal model experiment and the guiding principles in the use and care of laboratory animals were approved by The Second Ethical Committee on Animal Experiments (56/2013 and 54/2015). Maiden female Sprague-Dawley rats (*n* = 48, 30 days old) were purchased from Division of Experimental Animals, Department of General and Experimental Pathology (Medical University of Warsaw, Warsaw, Poland). During the whole experiment they were kept in an animal room at 21 °C, in a 12 h light:12 h dark cycle. Rats were fed ad libitum a standard laboratory fodder Labofeed H (Feed and Concentrates Production Plant, Poland). Fodder is mainly composed of 22.0% protein, 4.2% fat, 37.0% starch and 3.5% fiber with the minerals, vitamins and free amino acids constituting the other 33.3% of fodder. A detailed composition of Labofeed H fodder has been given previously [14].

After 1-week adaptation the animals were randomly divided into 4 groups of 12 individuals each. The detailed characteristics of experimental groups is given below:

CON—rats of the control group fed standard laboratory fodder and water ad libitum only,
PSO—rats with unlimited access to the standard laboratory fodder and water receiving PSO in the amount of 0.15 mL given via a gavage daily,
BME—rats with unlimited access to the standard laboratory fodder and 2% (*w*/*v*) BME as the only drinking liquid,
PSO + BME—rats fed a standard laboratory fodder and 2% (*w*/*v*) BME as the only drinking liquid and receiving PSO in the amount of 0.15 mL daily given via a gavage daily.

Food and fluid intake was monitored daily. BME intake given as the only drinking fluid in BME and PSO + BME groups was monitored daily and it did not differ from the water intake in CON and PSO groups. Supplementation of the diet was conducted for 21 weeks. In the 21st week of the experiment after 12 h of fasting, all animals were decapitated and exsanguinated.

### 4.4. Lipid Extraction and Sample Preparation for Fatty Acids Analysis in Liver

Samples of liver were thawed only once and lipids were extracted from three parallel samples of 0.2 g of hepatic tissue obtained from one animal, according to the procedure of Folch et al. with slight modification concerning the volumes of solvents [35,36]. Purified organic extracts obtained at the end of Folch procedure were evaporated to dryness under a stream of nitrogen. The fat content in hepatic tissue was estimated by weighing the dry residue. Obtained dry residue was taken for the preparation of FAME (fatty acid methyl ester). It was subjected to hydrolysis by heating with 2.5 mL of sodium methoxide in methanol (0.5 mol/L) at 80 °C for 10 min. Afterwards, fatty acids were converted to methyl esters by heating at 80 °C for 3 min with 2.5 mL of 14% boron trifluoride-methanol reagent. Isolation of obtained FAME with hexane (2 × 0.5 mL) was preceded by adding 1.0 mL of saturated sodium chloride solution. Hexane extracts were dried with anhydrous sodium sulfate and subsequently evaporated to dryness under a stream of nitrogen. FAME obtained were diluted in 20 μL of hexane and stored at −20 °C for chromatographic analysis.

### 4.5. Preparation of the Microsomal Fraction from Rat Liver

Hepatic microsomes were prepared according to the slightly modified method of Kłyszejko-Stefanowicz [24]. After its isolation, the liver was homogenized in 0.25 M sucrose buffered to pH 7.4, taking 16 mL buffer per every 4 g of the tissue. Next, it was rotated for 10 min at 1000× *g* in order to discard the cell debris, then the supernatant was again rotated for 20 min, at 16,000× *g*. After the mitochondrial precipitate was rejected, the remaining supernatant was rotated once more for 60 min, at 105,000× *g*. Then the precipitated microsomes were hung in 4 mL isolation medium. Thus, the prepared microsomal fraction was stored at the temperature of −70 °C until the time of carrying out of analysis.

### 4.6. Sample Preparation for Fatty Acids Analysis in Hepatic Microsomes

The hepatic microsomes were thawed only once and three parallel samples of 200 μL of microsomal suspensions from one animal were taken for lipids extraction. Briefly, a sample of microsomal suspension was mixed with 2.5 mL of chloroform: methanol (2:1, *v*/*v*) and after vigorous shaking the chloroform layer was separated. The residue was mixed with 1.5 mL of chloroform:methanol (2:1, *v*/*v*) and the extraction was repeated. The combined chloroform layers were centrifuged for 10 min at 1000× *g* and the precipitate was discarded. The organic extract was evaporated under stream of nitrogen and the residue was taken for the preparation of FAME according to procedure which was previously described for liver samples.

### 4.7. Instrumental Analysis

Samples of FAME from hepatic tissue and from hepatic microsomes were analyzed with gas chromatography (GC) with flame-ionization detection (FID). Gas chromatograph (GC-17A gas chromatograph, Shimadzu, Kyoto, Japan) equipped with capillary column (BPX 70; 60 m × 0.25 mm i.d., film thickness 0.20 μm, SGE, Ringwood, Australia) and Helium as a carrier gas was used. The temperature program of oven was: initially 140 °C for 1 min, increase by 20 °C/min to 200 °C, held for 20 min, increase by 5 °C/min to 220 °C and held for 25 min. The temperature of detector was 270 °C and the temperature of the injector was 270 °C. FAME standards (Supelco 37 Component FAME Mix) and CLA FAME reference standard (Nu-Chek-Prep, INC., Elysian, MN, USA) were used to identify the fatty acids present in the samples. The results were expressed as the percentage of individual fatty acid in total fatty acids’ pool.

Additionally, the peroxidability index (PI) of lipids was calculated on the basis of their FA composition, according to the following equation reported by Hsu et al. [37]:

PI = [(% dienoic × 1) + (% trienoic × 2) + (% tetraenoic × 3) + (% pentaenoic × 4) + (% hexaenoic × 5)](1)

### 4.8. Analysis of Δ^6^- and Δ^5^-Desaturase Activities

Estimation of ∆^6^- and Δ^5^-desaturase activities was done in accordance to the modified method outlined by Keelan et al. [38]. For further investigations, 0.2 mL of microsomes precipitate was taken and incubated in the reaction mixture. Each 1.0 mL of the reaction mixture consisted of 1.25 mM NADH; 2.25 mM glutathione; 5 mM ATP; 5 mM MgCl_2_; 0.1 mM CoA and 0.5 mM niacinamide, dissolved in buffer pH 7.4 and 200 nM linoleic acid sodium salt. The mixture was preincubated for 5 min at 37 °C. The reaction was initiated by adding 0.2 mL microsomes and incubating the mixture in a 90 min long shaking water bath at 37 °C. Further analytical procedure included extraction of lipids described by Folch et al. [39] and esterification [40]. High performance liquid chromatography with UV/VIS detector (Merck Hitachi) and YMC-Pack ODS-AM S-5 μm column was used to determine concentrations of linoleic (LA, C18:2 *n*-6 *cis*), γ-linolenic (GLA, C18:3 *n*-6), dihomo-γ-linolenic (DGLA, C20:3 *n*-6) and arachidonic acids. The column temperature was 30 °C and wavelength 198 nm.

The indices of D6D and D5D activity were determined as the ratio of GLA to the LA concentration in liver microsomes and the ratio of arachidonic acid (AA) concentration to the DGLA concentration, respectively. The differences in AA concentrations in incubated and non-incubated samples were also calculated, because the amount of AA synthesized in vitro from LA remained in close correlation with the activities of the investigated enzymes. AA levels were expressed in mg per 100 mg of microsomal protein (determined in accordance to the Lowry method [41]).

### 4.9. Measurement of Liver PGE_2_

PGE_2_ was measured in liver by a commercial enzyme immunoassay test kit (Prostaglandin E_2_ ELISA Kit-Monoclonal, Item No.514010, Cayman Chemical) according to manufacturer’s instructions. PGE_2_ concentrations in the samples were calculated from their corresponding absorbance values via the standard curve. The PGE_2_ level was expressed in pg/mg liver protein (protein determination was performed by Lowry protein assay [41]).

### 4.10. Statistical Analysis

All data are presented as means ± standard deviation. Statistica 12.5 software (StatSoft, Kraków, Poland) was used for the statistical analysis. For variables with normal distribution obtained data were tested with one-way ANOVA and post-hoc Tukey test (marked * in Tables). For variables without normal distribution the data were tested with Kruskal–Wallis test, which is a non-parametric equivalent of one-way ANOVA, with post-hoc multiple comparison test (marked ^#^ in Tables). The acceptable level of significance was established at *p* ≤ 0.05.

## 5. Conclusions

This is the first report demonstrating the influence of the separate and combined administration of PSO and BME on the composition and the metabolism of fatty acids in liver, in particular the activity of desaturases and the level of PGE_2_ in the liver and its microsomal fraction of rats. Applied dietary supplements exhibited an inhibiting effect on the activity of desaturases, and thus they contributed to the reduction in metabolites of AA. No significant intensification of the influence on the investigated parameters resulted from combined application of PSO and BME. A beneficial effect of dietary supplementation with BME and PSO on the formation of PGE_2_, which was found in the present work, can be one of the mechanisms of action of these botanicals. It is of utmost importance, as PGE_2_ is involved in the etiology of many diseases including different kinds of cancer. Obtained results revealed that PSO or BME could become helpful in the future prevention and treatment of many diseases. However, further research in this field seems to be desired and well founded.

## Figures and Tables

**Table 1:** Fatty acids profile [%] of BME and PSO.

Fatty Acids [%]:	BME	PSO
Lauric acid (C12:0)	nd	<0.1
Tridecanoic acid (C13:0)	1.3 ± 0.9	nd
Myristic acid (C14:0)	nd	0.3 ± 0.2
Pentadecanoic acid (C15:0)	0.4 ± 0.4	nd
Palmitic acid (C16:0)	17.4 ± 3.0	21.5 ± 0.3
Palmitoleic acid (C16:1)	nd	0.2 ± 0.0
Heptadecanoic acid (C17:0)	0.3 ± 0.1	0.4 ± 0.0
Heptadecenoic acid (C17:1)	nd	0.1 ± 0.0
Stearic acid (C18:0)	11.2 ± 2.6	17.7 ± 0.2
Oleic acid (C18:1 *n*-9 *cis*)	13.2 ± 3.1	10.9 ± 0.4
Linolelaidic acid (C18:2 *n*-6 *trans)*	nd	0.1 ± 0.0
Linoleic acid (C18:2 *n*-6 *cis*)	29.5 ± 7.5	20.0 ± 0.0
γ-linolenic acid (C18:3 *n*-6)	nd	0.1 ± 0.0
α-linolenic acid (C18:3 *n*-3)	11.2 ± 2.8	7.5 ± 0.1
Rumenic acid (C18:2 *cis*-9, *trans*-11)	nd	nd
Eicosenoic acid (C20:1)	nd	0.4 ± 0.0
Eicosadienoic acid (C20:2)	nd	0.3 ± 0.0
Docosanoic acid (C22:0)	nd	0.1 ± 0.0
Punicic acid (*cis*-9, *trans*-11, *cis*-13 C18:3)	nd	6.6 ± 0.2
*cis*-13,16-docosadienoic acid (C22:2)	nd	1.4 ± 0.2
Tricosylic Acid (C23:0)	nd	0.3 ± 0.0
Lignoceric acid (C24:0)	nd	0.1 ± 0.0

All data are shown as mean values ± standard deviation; BME—bitter melon extract; PSO—pomegranate seed oil; nd—not detected.

**Table 2:** Fatty acids profile in liver of experimental groups.

Fatty Acids [%]	CON (*n* = 12)	PSO (*n* = 12)	BME (*n* = 12)	PSO + BME (*n* = 12)	*p* Value
SFA					
Lauric acid (C12:0)	0.10 ± 0.10 ^a,b^	0.09 ± 0.06 ^b^	0.03 ± 0.01 ^a^	0.04 ± 0.01 ^a,b^	0.0052 ^#^
Tridecanoic acid (C13:0)	0.01 ± 0.01	0.05 ± 0.05	0.03 ± 0.03	0.01 ± 0.00	n.s.
Myristic acid (C14:0)	0.21 ± 0.11 ^b^	0.20 ± 0.07 ^b^	0.13 ± 0.02 ^a^	0.13 ± 0.01 ^a^	0.0017 ^#^
Pentadecanoic acid (C15:0)	0.22 ± 0.06 ^c^	0.15 ± 0.02 ^a^	0.17 ± 0.01 ^a,b,c^	0.17 ± 0.02 ^a,b^	0.0003 ^#^
Palmitic acid (C16:0)	17.8 ± 0.8	17.7 ± 0.9	17.6 ± 0.7	18.1 ± 0.8	n.s.
Heptadecanoic acid (C17:0)	0.70 ± 0.07 ^c^	0.56 ± 0.06 ^a^	0.64 ± 0.05 ^b,c^	0.61 ± 0.07 ^a,b^	<0.0001 *
Stearic acid (C18:0)	22.0 ± 1.7	22.7 ± 1.3	22.6 ± 1.4	23.0 ± 1.3	n.s.
Heneicosanoic acid (C21:0)	0.16 ± 0.03 ^c^	0.14 ± 0.02 ^a^	0.14 ± 0.01 ^a,b^	0.15 ± 0.03 ^a,b,c^	0.0133 ^#^
Docosanoic acid (C22:0)	0.02 ± 0.01	0.02 ± 0.01	0.02 ± 0.01	0.02 ± 0.01	n.s.
Tricosylic Acid (C23:0)	0.05 ± 0.03	0.04 ± 0.01	0.03 ± 0.01	0.03 ± 0.02	n.s.
Lignoceric acid (C24:0)	0.15 ± 0.03	0.09 ± 0.07	0.17 ± 0.03	0.16 ± 0.02	n.s.
Σ SFA	41.3 ± 1.2	41.8 ± 0.7	41.6 ± 1.3	42.4 ± 0.9	n.s.
MUFA					
Pentadecenoic acid (C15:1)	0.02 ± 0.01	0.02 ± 0.01	0.02 ± 0.00	0.02 ± 0.00	n.s.
Palmitoleic acid (C16:1)	0.72 ± 0.23	0.68 ± 0.10	0.69 ± 0.17	0.64 ± 0.13	n.s.
Heptadecenoic acid (C17:1)	0.13 ± 0.01 ^b^	0.11 ± 0.01 ^a,b^	0.12 ± 0.02 ^a,b^	0.11 ± 0.02 ^a^	0.0242 *
Elaidic acid (C18:1 *n*-9 *trans*)	0.20 ± 0.07 ^b^	0.10 ± 0.00 ^a^	0.18 ± 0.05 ^b^	0.18 ± 0.04 ^b^	0.0156 ^#^
Oleic acid (C18:1 *n*-9 *cis*)	7.75 ± 2.05	6.65 ± 0.69	7.17 ± 1.24	6.1 ± 1.0	n.s.
Eicosenoic acid (C20:1)	0.08 ± 0.01	0.09 ± 0.02	0.08 ± 0.03	0.07 ± 0.04	n.s.
Docosenoic acid (C22:1)	0.01 ± 0.00	0.01 ± 0.00	0.02 ± 0.00	0.02 ± 0.01	n.s.
Σ MUFA	8.88 ± 2.25	7.59 ± 0.76	8.27 ± 1.43	7.08 ± 1.16	n.s.
PUFA					
Linolelaidic acid (C18:2 *n*-6 *trans*)	0.15 ± 0.08 ^c^	0.11 ± 0.06 ^a,b,c^	0.07 ± 0.02 ^a,b^	0.06 ± 0.02 ^a^	0.0228 ^#^
Linoleic acid (C18:2 *n*-6 *cis*)	15.0 ± 1.3	15.5 ± 0.9	15.5 ± 0.9	15.0 ± 1.4	n.s.
γ-linolenic acid (C18:3 *n*-6)	0.22 ± 0.06 ^a^	0.31 ± 0.06 ^c^	0.28 ± 0.09 ^a,b,c^	0.23 ± 0.04 ^a,b^	0.0013 *
α-linolenic acid (C18:3 *n*-3)	1.16 ± 0.24	1.24 ± 0.18	1.17 ± 0.22	1.09 ± 0.22	n.s.
Rumenic acid (C18:2 *cis*-9, *trans*-11)	0.037 ± 0.007 ^a^	0.34 ± 0.08 ^b^	0.041 ± 0.008 ^a^	0.41 ± 0.14 ^b^	<0.0001 ^#^
Eicosadienoic acid (C20:2)	0.11 ± 0.06 ^b^	0.07 ± 0.03 ^a,b^	0.07 ± 0.02 ^a,b^	0.05 ± 0.02 ^a^	0.0224 ^#^
Dihomo-γ-linolenic acid (C20:3 *n*-6)	0.47 ± 0.04 ^a,b^	0.32 ± 0.22 ^a^	0.52 ± 0.08 ^b^	0.53 ± 0.09 ^b^	0.0188 ^#^
Arachidonic acid (C20:4 *n*-6)	16.2 ± 1.4 ^a^	18.5 ± 0.8 ^b^	17.8 ± 1.4 ^b^	18.3 ± 1.1 ^b^	<0.0001 *
Eicosatrienoic Acid (C20:3 *n*-3)	0.16 ± 0.03 ^a^	0.16 ± 0.02 ^a,b^	0.18 ± 0.04 ^a,b,c^	0.21 ± 0.07 ^c^	0.0066 ^#^
Eicosapentaenoic acid (C20:5 *n*-3)	0.82 ± 0.14	0.87 ± 0.15	0.89 ± 0.17	0.84 ± 0.19	n.s.
*cis*-13,16-docosadienoic acid (C22:2)	0.03 ± 0.00	0.04 ± 0.01	0.03 ± 0.02	0.05 ± 0.02	n.s.
Docosahexaenoic acid (C22:6 *n*-3)	9.41 ± 1.29 ^b^	8.08 ± 0.88 ^a^	8.35 ± 1.04 ^a,b^	8.85 ± 0.84 ^a,b^	0.0152 *
Σ PUFA	43.5 ± 2.1 ^a^	45.5 ± 1.0 ^b^	44.9 ± 0.9 ^a,b^	45.5 ± 0.62 ^b^	0.0072 ^#^
*n*-3	11.5 ± 1.1 ^b^	10.3 ± 0.8 ^a^	10.6 ± 0.9 ^a,b^	11.0 ± 0.6 ^a,b^	0.0102 *
*n*-6	31.8 ± 1.5 ^a^	34.7 ± 0.8 ^b^	34.2 ± 1.2 ^b^	34.0 ± 0.6 ^b^	0.0001 ^#^
*n*-6/*n*-3	2.78 ± 0.25 ^a^	3.37 ± 0.28 ^b^	3.26 ± 0.39 ^b^	3.10 ± 0.2 ^b^	<0.0001 *
(MUFA + PUFA)/SFA	1.27 ± 0.07	1.27 ± 0.04	1.28 ± 0.06	1.24 ± 0.05	n.s.
PUFA/SFA	1.05 ± 0.06	1.09 ± 0.03	1.08 ± 0.03	1.07 ± 0.03	n.s.
PI	163.9 ± 13.8	164.1 ± 6.8	163.6 ± 7.9	168.7 ± 7.3	n.s.

All data are shown as mean values ± standard deviation. Three parallel samples were prepared from the experimental material obtained from each of twelve animals in each experimental group. *p* value ≤ 0.05—significant differences among groups in one-way ANOVA (*) or Kruskal–Wallis test (#). Values with a different index in one row are significantly different from each other (*p* ≤ 0.05) in post-hoc RIR Tukey test or multiple comparison test. n.s.—not significant (*p* > 0.05); CON—control group; PSO—group receiving PSO in the amount of 0.15 mL/day; BME—group receiving BME ad libitum; PSO + BME—group receiving PSO in the amount of 0.15 mL/day and BME ad libitum; MUFA—monounsaturated fatty acids; PUFA—polyunsaturated fatty acids; SFA—saturated fatty acids; PI—peroxidability index; Σ—sum of SFA, MUFA or PUFA, respectively.

**Table 3:** Fatty acids profile in hepatic microsomes of experimental groups.

Fatty Acids [%]:	CON (*n* = 12)	PSO (*n* = 12)	BME (*n* = 12)	PSO + BME (*n* = 12)	*p* Value
SFA					
Lauric acid (C12:0)	0.07 ± 0.04 ^a,b^	0.07 ± 0.01 ^b^	0.09 ± 0.04 ^b^	0.05 ± 0.02 ^a^	0.0058 ^#^
Tridecanoic acid (C13:0)	0.03 ± 0.02	0.03 ± 0.01	0.03 ± 0.02	0.04 ± 0.02	n.s.
Myristic acid (C14:0)	0.24 ± 0.06	0.21 ± 0.03	0.20 ± 0.03	0.20 ± 0.04	n.s.
Pentadecanoic acid (C15:0)	0.23 ± 0.05 ^b^	0.17 ± 0.02 ^a^	0.19 ± 0.02 ^a,b^	0.19 ± 0.03 ^a,b^	0.0036 ^#^
Palmitic acid (C16:0)	18.2 ± 0.8	18.2 ± 1.6	17.5 ± 0.9	18.0 ± 1.0	n.s.
Heptadecanoic acid (C17:0)	0.72 ± 0.06 ^c^	0.56 ± 0.06 ^a^	0.64 ± 0.05 ^b^	0.58 ± 0.06 ^a,b^	<0.0001 *
Stearic acid (C18:0)	24.4 ± 1.9	24.8 ± 1.6	24.2 ± 1.9	23.7 ± 0.9	n.s.
Heneicosanoic acid (C21:0)	0.10 ± 0.02	0.08 ± 0.01	0.10 ± 0.02	0.09 ± 0.02	n.s.
Docosanoic acid (C22:0)	0.03 ± 0.01	0.03 ± 0.01	0.04 ± 0.01	0.04 ± 0.01	n.s.
Tricosylic Acid (C23:0)	0.03 ± 0.00 ^a^	0.06 ± 0.03 ^a,b^	0.05 ± 0.02 ^a,b^	0.08 ± 0.04 ^b^	0.0059 ^#^
Lignoceric acid (C24:0)	0.19 ± 0.06	0.19 ± 0.03	0.18 ± 0.02	0.17 ± 0.03	n.s.
Σ SFA	44.2 ± 1.9	44.4 ± 1.6	43.2 ± 1.4	43.1 ± 1.1	n.s.
MUFA					
Myristoleic acid (C14:1)	0.03 ± 0.01 ^b^	0.01 ± 0.01 ^a,b^	0.01 ± 0.01 ^a^	0.02 ± 0.01 ^a,b^	0.0119 ^#^
Pentadecenoic acid (C15:1)	0.02 ± 0.01	0.02 ± 0.01	0.02 ± 0.00	0.03 ± 0.03	n.s.
Palmitoleic acid (C16:1)	0.65 ± 0.13	0.59 ± 0.13	0.58 ± 0.13	0.63 ± 0.13	n.s.
Heptadecenoic acid (C17:1)	0.12 ± 0.01 ^b^	0.10 ± 0.02 ^a^	0.12 ± 0.02 ^a,b^	0.11 ± 0.02 ^a,b^	0.0147 ^#^
Elaidic acid (C18:1 *n*-9 *trans*)	0.23 ± 0.08 ^b^	0.14 ± 0.07 ^a^	0.18 ± 0.04 ^a,b^	0.19 ± 0.04 ^a,b^	0.0080 *
Oleic acid (C18:1 *n*-9 *cis*)	7.68 ± 1.25 ^c^	5.92 ± 0.80 ^a^	6.96 ± 0.83 ^a,b,c^	6.07 ± 0.92 ^a,b^	0.0007 ^#^
Eicosenoic acid (C20:1)	0.10 ± 0.01 ^b^	0.08 ± 0.02 ^a,b^	0.09 ± 0.03 ^b^	0.06 ± 0.02 ^a^	0.0010 ^#^
Σ MUFA	8.78 ± 1.32 ^c^	6.85 ± 0.92 ^a^	7.96 ± 0.97 ^a,b,c^	7.07 ± 1.05 ^a,b^	0.0009 ^#^
PUFA					
Linolelaidic acid (C18:2 *n*-6 *trans*)	0.07 ± 0.03	0.06 ± 0.02	0.07 ± 0.02	0.06 ± 0.02	n.s.
Linoleic acid (C18:2 *n*-6 *cis*)	13.1 ± 1.0	13.3 ± 0.9	13.2 ± 1.0	12.8 ± 1.5	n.s.
γ-linolenic acid (C18:3 *n*-6)	0.18 ± 0.03 ^a^	0.27 ± 0.08 ^c^	0.26 ± 0.07 ^b,c^	0.20 ± 0.04 ^a,b^	0.0002 ^#^
α-linolenic acid (C18:3 n-3)	1.09 ± 0.20	1.05 ± 0.20	1.03 ± 0.17	1.10 ± 0.21	*n*.s.
Rumenic acid C18:2 (*cis*-9, *trans*-11)	0.052 ± 0.011 ^a^	0.31 ± 0.05 ^b^	0.049 ± 0.009 ^a^	0.34 ± 0.09 ^b^	<0.0001 ^#^
Eicosadienoic acid (C20:2)	0.12 ± 0.08 ^b^	0.06 ± 0.03 ^a,b^	0.08 ± 0.02 ^b^	0.05 ± 0.02 ^a^	0.0040 ^#^
Dihomo-γ-linolenic acid (C20:3 *n*-6)	0.46 ± 0.09	0.41 ± 0.11	0.48 ± 0.08	0.45 ± 0.09	n.s.
Arachidonic acid (C20:4 *n*-6)	16.9 ± 1.1 ^a^	20.4 ± 1.3 ^c^	19.8 ± 1.2 ^b,c^	19.0 ± 1.4 ^b^	<0.0001 *
Eicosatrienoic Acid (C20:3 *n*-3)	0.28 ± 0.11	0.28 ± 0.05	0.24 ± 0.03	0.28 ± 0.05	n.s.
Eicosapentaenoic acid (C20:5 *n*-3)	0.72 ± 0.15	0.80 ± 0.17	0.85 ± 0.16	0.74 ± 0.16	n.s.
*cis*-13,16-docosadienoic acid (C22:2)	0.05 ± 0.04	0.03 ± 0.02	0.04 ± 0.02	0.05 ± 0.03	n.s.
Docosahexaenoic acid (C22:6 *n*-3)	8.27 ± 0.91	7.73 ± 1.01	8.10 ± 1.25	8.14 ± 0.98	n.s.
Σ PUFA	41.2 ± 2.1 ^a^	44.7 ± 1.4 ^b^	44.1 ± 1.2 ^b^	43.2 ± 1.5 ^a,b^	0.0003 ^#^
*n*-3	10.3 ± 1.0	9.85 ± 0.86	10.2 ± 1.2	10.2 ± 0.7	n.s.
*n*-6	30.6 ± 1.5 ^a^	34.4 ± 1.3 ^c^	33.7 ± 0.9 ^b,c^	32.6 ± 1.2 ^b^	<0.0001 *
*n*-6/*n*-3	2.98 ± 0.23 ^a^	3.52 ± 0.34 ^b^	3.35 ± 0.47 ^b^	3.19 ± 0.23 ^a,b^	0.0023 *
(MUFA + PUFA)/SFA	1.13 ± 0.09	1.16 ± 0.08	1.21 ± 0.07	1.17 ± 0.05	n.s.
PUFA/SFA	0.93 ± 0.08 ^a^	1.01 ± 0.07 ^b^	1.02 ± 0.05 ^b^	1.00 ± 0.05 ^b^	0.0055 *
PI	155.5 ± 10.1 ^a^	166.2 ± 9.2 ^b^	166.6 ± 8.9 ^b^	163.1 ± 10.7 ^a,b^	0.0271 *

All data are shown as mean values ± standard deviation. Three parallel samples were prepared from experimental material obtained from each of twelve animals in each experimental group. *p* value ≤ 0.05 - significant differences among groups in one-way ANOVA (*) or Kruskal–Wallis test (#). Values with a different letter index in one row are significantly different from each other (*p* ≤ 0.05) in post-hoc RIR Tukey test or multiple comparison test. n.s.—not significant (*p* > 0.05); CON—control group; PSO—group receiving PSO in the amount of 0.15 mL/day; BME—group receiving BME ad libitum; PSO + BME—group receiving PSO in the amount of 0.15 mL/day and BME ad libitum; MUFA—monounsaturated fatty acids; PUFA—polyunsaturated fatty acids; SFA—saturated fatty acids; PI—peroxidability index; Σ—sum of SFA, MUFA or PUFA, respectively.
